# A dual larynx motor networks hypothesis

**DOI:** 10.1098/rstb.2020.0392

**Published:** 2021-11-01

**Authors:** Michel Belyk, Nicole Eichert, Carolyn McGettigan

**Affiliations:** 1 Department of Speech Hearing and Phonetic Sciences, University College London, London, United Kingdom; 2 Wellcome Centre for Integrative Neuroimaging, Centre for Functional MRI of the Brain (FMRIB), Nuffield Department of Clinical Neurosciences, John Radcliffe Hospital, University of Oxford, Oxford, United Kingdom

**Keywords:** larynx, somatotopy, motor system, brain evolution, cerebellum, supplementary motor area

## Abstract

Humans are vocal modulators par excellence. This ability is supported in part by the dual representation of the laryngeal muscles in the motor cortex. Movement, however, is not the product of motor cortex alone but of a broader motor network. This network consists of brain regions which contain somatotopic maps that parallel the organisation in motor cortex. We therefore present a novel hypothesis that the dual laryngeal representation is repeated throughout the broader motor network. In support of the hypothesis we review existing literature which demonstrates the existence of network-wide somatotopy, and present initial evidence for the hypothesis’ plausibility. Understanding how this uniquely human phenotype in motor cortex interacts with broader brain networks is an important step toward understanding how humans evolved the ability to speak. We further suggest that this system may provide a means to study how individual components of the nervous system evolved within the context of neuronal networks.

Humans are vocal modulators par excellence. This is usually characterised as the capacity for Vocal Production Learning (VPL), which is the ability to learn to produce novel vocalisations [1]. Few species of mammals, such as cetaceans and bats [2,3], have displayed strong VPL abilities, and none of these species has a close phylogenetic relationship to humans. Monkeys are particularly weak vocal learners [4]. Non-human apes appear to have intermediate VPL, being able to learn certain kinds of limited vocal behaviour from humans [5,6], though there is little evidence of this behaviour in the wild [7]. The human VPL capacity is attributable in part to specialised adaptations in motor cortex that grant voluntary control over the voice. However, complex behavioural abilities such as VPL are not the product of the motor cortex alone but are an emergent property of their interaction with a broader motor network.

Human motor cortex is composed of a band of specialized grey matter along the precentral gyrus and the anterior bank of the precentral sulcus, which is the main source of motor output from the central nervous system. Penfield’s seminal neurosurgical studies [8] described the conspicuous somatotopy of the human primary motor cortex (M1), in which the muscles of the foot are represented at one end of the somatotopic map and the muscles of the head represented at the other end [9–11]. Similar somatotopic maps have been described throughout the network of brain areas that control movement, including the cerebellum, supplementary motor area (SMA), basal ganglia (BG), and the middle cingulate cortex (MCC) [12–15].

Penfield’s original mapping was uncertain of the somatotopic location of the laryngeal muscles, which control the sound source of the voice. More recent neurosurgical [16,17], molecular genetic [18], and brain imaging studies [19–24] provide compelling evidence that the laryngeal muscles are unusual in being controlled by two distinct loci within the human motor cortex. While other effectors such as the digits of the hand may also have multiple representations in motor cortex, these tend to be contiguous and may represent either subdivision at a finer scale (i.e., muscles of flexion vs. extension) or correlated movements with nearby muscles that exert a common influence over shared joints [25–28]. In contrast, the dual laryngeal representations are non-contiguous, being located at opposing ends of the orofacial motor zone - which is a marked deviation from the single larynx area observed in other primates [29,30]. The two representations have therefore been referred to as dorsal and ventral laryngeal motor cortex (dLMC, vLMC). This adaptation has clear implications for the evolution of speech since the neural control of the larynx supports one of the requirements of spoken language [31,32], namely a high degree of control over the voice source beyond the capabilities of other primates [4,33].

Despite extensive searches spanning new world monkeys (primarily *Macaca mulatta*), old world monkeys (primarily *Saimiri sciureus*), and all extant genera of great apes including Chimpanzees (*Pan troglodytes*), Orangutans (*Pongo* sp.), and Gorillas (*Gorilla* sp.) [29,34,35], humans appear to be the sole primate with the neural trait of dual larynx representation, and much has been written about the possible implications of this phenotype for the evolution of speech [36–41]. Here, we outline a novel hypothesis that this human phenotype is not restricted to the motor cortex but extends throughout a network of somatotopically-arranged brain areas that comprise the motor system, including the cerebellum, SMA, BG, and MCC and the axonal projections between these regions.

## Hypothesis: Dual larynx motor networks

**Figure 1:**
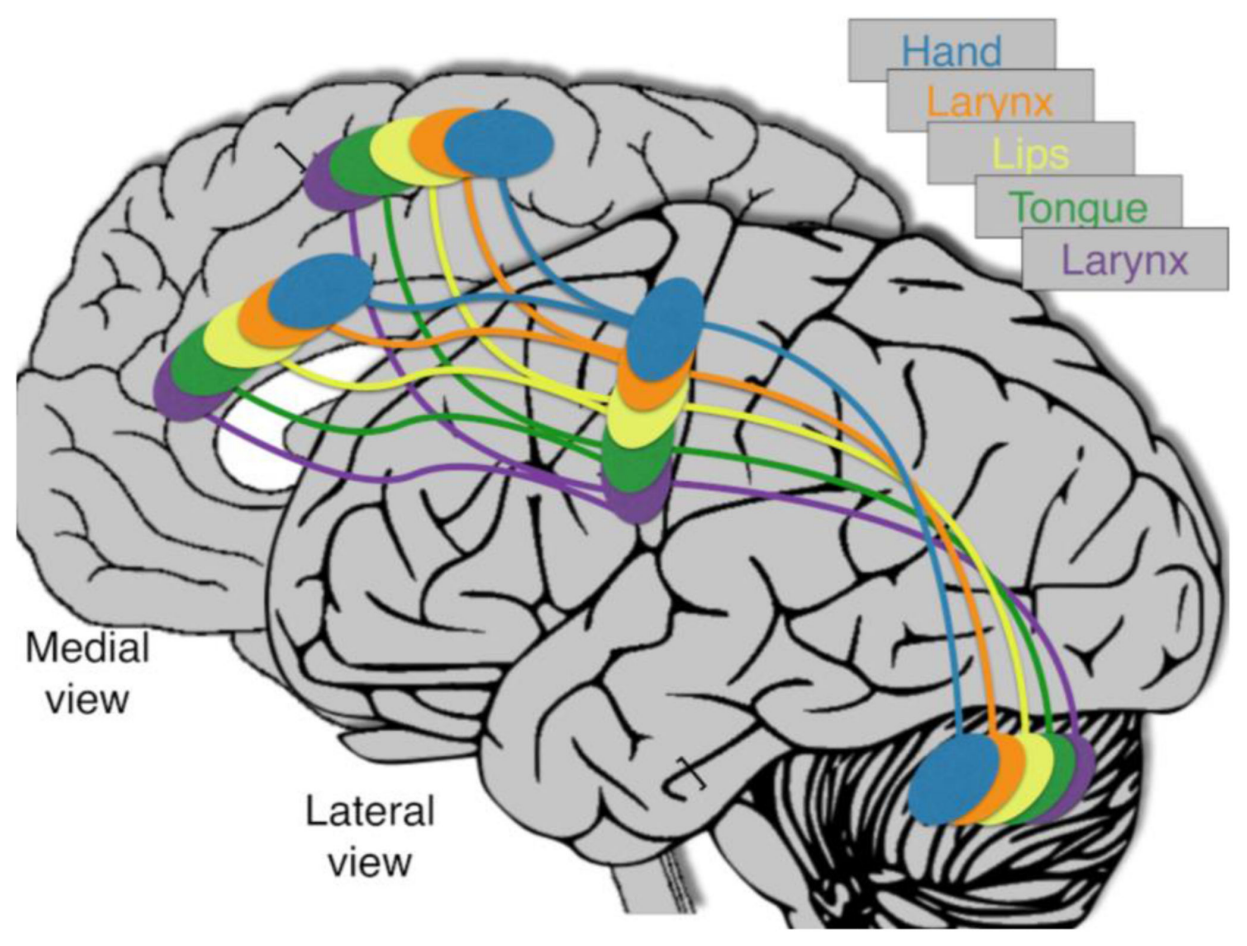
Depiction of the dual laryngeal motor network hypothesis. The middle cingulate cortex, supplementary motor area, and cerebellum are depicted with simplified somatotopic maps for conceptual convenience. The broader motor somatotopy follows the organisation of motor cortex, but with idiosyncratic orientations following a different axis in each brain region (basal ganglia not shown for simplicity). The hypothesised dLMC-related and vLMC-related networks are shown in orange and purple, respectively.

We hypothesize that each motor region contains two representations of the laryngeal muscles within their respective somatotopic maps: one between the hand and the orofacial muscles, and a second at the end of the orofacial representation (see Figure 1). This hypothesis is supported by the observations that i) somatotopic maps throughout the motor network follow a similar ordering of representations from foot to face and ii) nodes in the motor network project to one another homotopically, suggesting that motor regions beyond motor cortex must have target zones that receive the projections from the dLMC and vLMC. Somatotopic maps in different regions vary in orientation. For instance, somatotopy proceeds dorso-ventrally in the motor cortex but antero-posteriorly along the medial wall. Therefore, it may not be constructive to use the labels dorsal and ventral larynx areas for somatotopic maps beyond motor cortex. We have therefore adopted the convention of referring to larynx somatotopic regions in the MCC, SMA, cerebellum, and BG as dLMC-related or vLMC-related to denote their respective positions within the somatotopically arranged motor network.

An alternative hypothesis is that only the dLMC benefits from the gain in function concomitant with support from the broader motor system. Only dLMC is composed of primary motor cortex, while vLMC is likely to be located in a qualitatively different cytoarchitectonic motor region (see a more detailed discussion below). Moreover, dLMC is a novel phenotype in humans and robustly observed in human functional brain imaging studies, which points towards a prominent role in brain architecture. Therefore, if only one larynx representation is observed in the network of somatotopic maps, , then we predict that it will be the dLMC-related locus in a position between the hand and the articulatory muscles. If this turns out to be the case, it will regardless be important to understand the evolution of the dLMC in the context of a broader motor network.

## A human-specific phenotype in motor cortex

Compared to other primates, lower motor neurons in the human spinal cord and brainstem receive a far greater proportion of their inputs from neocortex. These connections contribute to the dexterity and behavioural flexibility of our species [42–44]. Included in this abundance of cortical efferents is a direct projection to motor neurons in the nucleus ambiguus [36–40], which is a brainstem motor nucleus that controls the muscles of the larynx. Such a direct cortico-bulbar connection is lacking in monkeys [45], extant but sparse in non-human apes [35], and further elaborated in humans [46,47]. An analogous phenotype distinguishes birds who are strong vocal learners such as songbirds (*order Passeriformes*), humming birds (*order Apodiformes*), and parrots (*order Psitaciformes*) from weaker vocal learners [48,49]. Thus, it appears that multiple phylogenetic lineages with strong VPL abilities have converged on similar neurophenotypes with direct efferent projection from upstream motor areas to voice-motor nuclei [50,51].

Evidence for the presence of this direct connection between the neocortex and the nucleus ambiguus in humans has come from natural experiments due to cerebrovascular events [46,47], in which large cortical lesions caused the axons of upper motor neurons to degenerate. Tracing the course of these damaged axons against the more intact surrounding white matter allowed the authors to demonstrate the existence of the direct cortico-bulbar pathway. However, these lesions all resulted from cerebrovascular accidents of the middle cerebral artery (MCA) that can result in widespread damage across the speech relevant portions of motor cortex (hence the prevalence of speech-motor and swallowing disorders following MCA infarcts; [52,53]). Thus, lesion studies provide limited information about the cortical source of the direct pathway.

Researchers using functional neuroimaging to investigate speech motor control initially presumed that the larynx was represented at the ventral-most extent of primary motor cortex [54], in the location that would be expected from the larynx’s position within the throat and proximity to the homologous region in non-human primates [29,55,56]. However, later studies demonstrated that the human brain in fact has two separate representations of the larynx, at either end of the orofacial somatotopic map of the precentral gyrus [19–23]. Though the dual larynx representations have not been consistently labelled as such in earlier brain imaging research, it was nonetheless consistently present near the predicted location [54].

The dLMC is located in canonical primary motor cortex in Brodmann Area (BA) 4, which is cytoarchitecturally defined as the region containing a high abundance of giant pyramidal neurons in cortical layer V - these pyramidal neurons are the source of the descending motor pathways of the cortico-spinal and cortico-bulbar tracts [57–59].

In contrast, the human vLMC is localized to the most ventral segment of the central sulcus or the lateral segment of the anterior subcentral sulcus [17,18,60]. The localisation of the vLMC may be particularly variable due to a high degree of individual variation in the morphology of nearby sulci [60], which may explain why the vLMC escaped notice by many early functional magnetic resonance imaging (fMRI) studies. Unlike its dorsal counterpart, quantitative neuroimaging has also suggested that the vLMC is not located in primary motor cortex [60]. Although no study has both localized the vLMC and performed a cytoarchitectural analysis of the underlying tissue, the location of the vLMC corresponds to BA 43 in the Brodmann atlas. While Brodmann believed that this region most strongly resembled somatosensory cortex based on its cellular composition [57], Vogt believed that it more strongly resembled motor cortex based on the degree of myelination of cortical layer V, which is an indicator of the large myelinated axons that form the efferent motor pathways that carry motor commands to the peripheral nervous system [58,59]. In contrast to the evidence from humans, the larynx representation in non-human primates has been identified in premotor cortex [45], but no separate representation in primary motor cortex has been described. This observation is in line with the theory that primary and premotor cortex contain one single somatotopic map spanning cytoarchitectural zones [61].

Whether the dLMC and vLMC make separate functional contributions to voice motor control, and what those might be, remains an active area of research. Identifying behaviours that activate one of these regions over the other is challenging, given that the dLMC may be easier to detect than the vLMC. However, electrical stimulation studies in humans have observed that stimulation of the dLMC elicits a vowel-like vocalisation, while stimulation of the vLMC elicits grunting [8,16,62]. The dLMC is bounded posteriorly by a putative larynx sensory cortex on the posterior central gyrus. This Larynx Sensory Cortex (LSC) is larger and activates more strongly in professional Opera singers than non-singers, suggesting that these individuals make greater use of proprioceptive feedback to guide highly skilled motor control [63,64].

It is not clear whether the vLMC is bounded posteriorly by a sensory zone, analogous to the dLMC. However, the vLMC may itself have some sensory function not matched by its dorsal counterpart. While the vLMC has primarily been localized as a correlate of vocal motor behaviour [17,20–23], activation of this region has also been observed in response to sensory stimulation of the larynx by applying an external puff of air [65]. Somewhat paradoxically, anesthetising the larynx does not reduce vLMC activation [19]. A recent cortical parcellation based on multi-modal brain imaging confirms that this region is distinct from both primary motor and primary somatosensory cortex and suggests a combination of sensorimotor functions [66]. Further research on the relationship between the vLMC and the broader motor system may shed further light on its function.

## The motor system and its somatotopic maps

**Figure 2:**
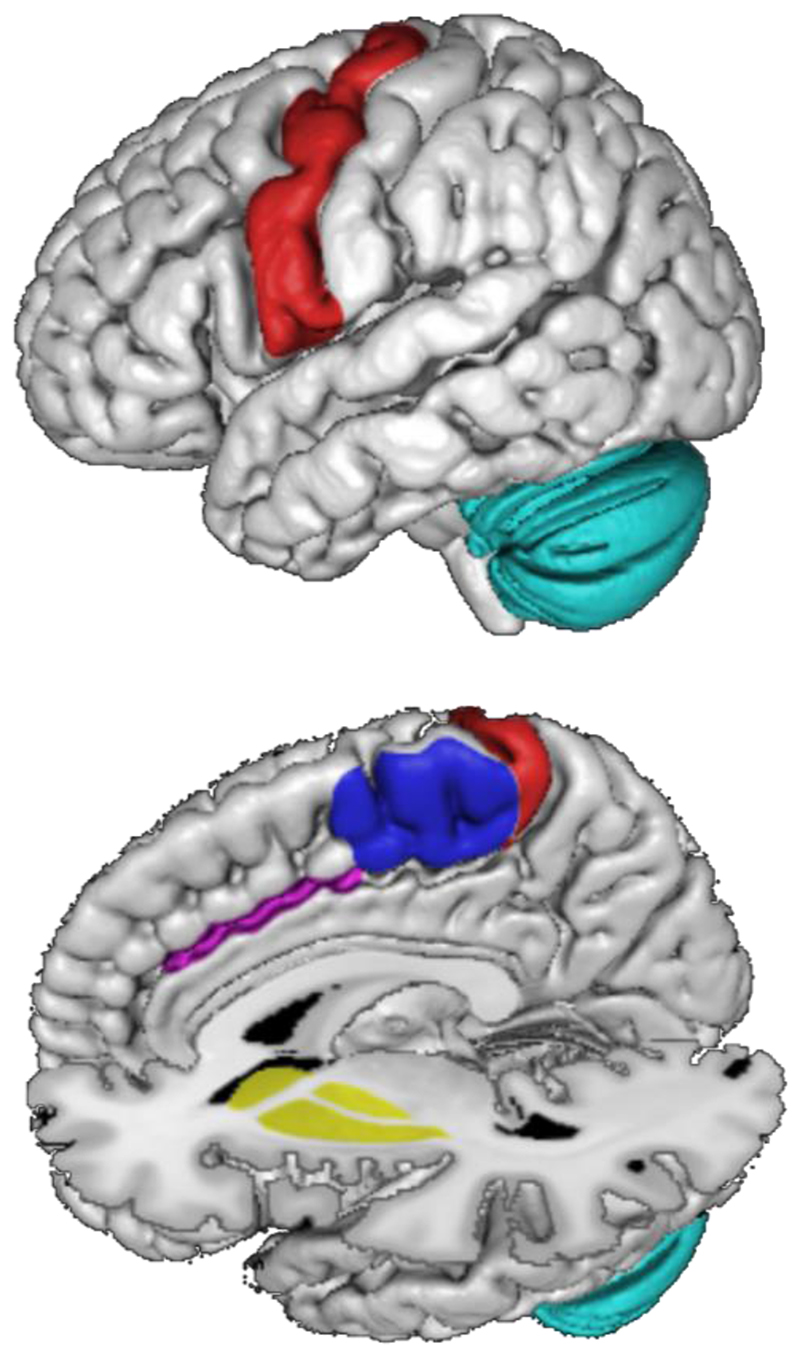
Major components of the motor network. A) Lateral surface view of MNI152 atlas brain, b) medial surface view with digital transections at x=0 and z=0 showing the motor cortex (red), middle cingulate cortex, (Pink), basal ganglia (yellow), supplementary motor area (blue), and cerebellum (cyan).

Motor cortex is the main source of output from the motor system. However, motor control is not the product of M1 alone, but requires a broader motor network that supports complex voluntary movements. This network includes brain regions such as the basal ganglia, supplementary motor area (SMA), cingulate cortex, and the cerebellum (See Figure 2). In this section we review the existing evidence that each of these brain regions contains its own somatotopic map akin to motor cortex. Intriguingly, the somatotopic maps in the brains of individuals born without one hand undergo a neuroplastic remapping that may occur in parallel across multiple brain regions within this network [67], which may suggest that somatotopic maps across the motor network are driven by common developmental mechanisms.

### Motor Cortex

The somatotopic map in primary motor cortex (BA 4) is well characterized and is sometimes referred to as a homunculus in the brain after its reflection of the physical body. The muscles of the foot are located at one end of the somatotopic map and the muscles of the head located at the other [9–11]. For conceptual convenience, zones within these somatotopic maps are often referred to by simplistic labels based on the effectors with which they are most strongly associated (e.g., M1_hand_ for the predominantly hand controlling zone). However, at a finer spatial scale these zones are composed of tessellated fields and individual effectors can be controlled by discontinuous but clustered representations [68]. These representations have been described as either encoding the states of muscles [69,70], the spatial properties of movement vectors [71,72], or ethologically meaningful combinations of effectors that pattern whole movements [61,73]. These levels of encoding are not mutually exclusive [74].

Distinct functional contributions of the dLMC and vLMC remain elusive [75,76]. However, electrical stimulation of these regions in the human brain elicit vowel sounds and grunting, respectively [8,16,62]. These separate behaviours produced by the same ensemble of muscles is suggestive of distinct ethological functions of the dLMC and vLMC, though further evidence is required. It is hoped that an understanding of the connections of these two regions with the broader motor system will begin to elucidate their respective functions.

### Cortico-cerebellar loops

The cerebellum maintains a broad pattern of connections throughout the brain and has some part in a wide range of central nervous system function [77,78]. Among these functions the cerebellum plays a critical role in making online adjustments that fine-tune movements. The cerebellum receives an efferent copy of motor commands from M1 and compares expected proprioceptive feedback with observed proprioceptive feedback [79–82]. The difference between intended and observed movements produces an error signal that is returned to M1 to implement online corrections to ongoing movements.

The cerebellum contains at least two separate somatotopic maps [83]. The anterior lobe of the cerebellum contains a somatotopic map with the foot located antero-dorsally and the head postero-ventrally, while the posterior lobe has a somatotopic map with the face represented postero-dorsally and the foot antero-ventrally [84–88]. More recent evidence suggests that the anterior lobe may contain an additional somatotopic map along lateral-to-medial axis [89], though further replication is required.

### Cortico-striatal loops

The supplementary motor area and basal ganglia form part of the cortico-striatal loop which is involved in motor learning [90,91]. The motoric processing loop of the basal ganglia forms a circuit through its various component nuclei including the putamen (a part of the striatum for which this circuit is named), globus pallidus, subthalamic nucleus, and substantia nigra, which sends outputs via the thalamus back to the cortex [92]. This circuit receives dopaminergic inputs from reward centres to mediate reinforcement learning [93,94].

The SMA and a region anterior to it called the pre-SMA both contain a distinct set of motor representations, with a clear somatotopy at least in SMA (Picard and Strick 1996). This somatotopic map spans from the legs posteriorly to the orofacial muscles anteriorly [13,95–98]. The putamen receives inputs from both M1 and the SMA and these inputs retain the somatotopic organization of their sources [15]. Inputs from M1 and the SMA innervate distinct portions of the putamen and it has therefore been suggested that the putamen may contain two parallel somatotopic maps [99]. Somatotopy may also be retained throughout the entire cortico-striatal loop [100], including the globus pallidus [101,102] and thalamus [103] though on a spatial scale that is inaccessible to current non-invasive brain imaging methodologies.

### Cingulate cortex

The cingulate cortex is nested in the medial surface of the brain following the curvature of the corpus callosum. This brain region combines cognitive, affective, and motoric functions for the motivation and initiation of goal-directed behaviours [104–107]. It is divided grossly into the anterior, middle, and posterior cingulate cortex (ACC, MCC and PCC, respectively). The MCC has approximate boundaries anteriorly at the genu of the corpus callosum and posteriorly at the marginal sulcus [108–110]. This macro-anatomically defined region itself comprises multiple cytoarchitecturally defined subregions. Of these, area 24c is in the cingulate sulcus, which contains a series of three cingulate motor areas [12,111]. These cingulate motor areas are all involved in action selection, with increasingly more complex movement patterns involving the more anterior divisions [112–114].

The middle cingulate sulcus contains three distinct motor regions [12,111] each of which contains a somatotopic map with the feet represented posteriorly and the orofacial muscles anteriorly [12,114–118]. Somatotopic mapping in the cingulate cortex may be further complicated by the high degree of anatomical variability of this region, since in a subset of human brains the motor regions of the cingulate sulcus are divided across separate cingulate and paracingulate sulci [12,119–121].

### White matter somatotopy

The descending motor pathways which form the corticobulbar and corticospinal outputs from the motor system maintain a clear somatotopic map that is observable in white matter [122–125]. This somatotopy facilitates the mapping of upper motor neurons in primary motor cortex onto their corresponding lower motor neurons in the brainstem and spinal cord. Likewise, the somatotopic maps of M1 in either hemisphere project preferentially to homotopic sites in the opposite hemisphere, retaining ordered somatotopy in the white matter of the corpus callosum [126,127]. At least some of the individual brain regions that make up the motor network also display preferential functional connectivity between somatotopically analogous regions [87,128], maintaining somatotopy in the white matter pathways that connect them [115,129].

## Initial evidence for dual laryngeal representations in the cerebellum and SMA

### Cerebellum

We re-analysed an existing fMRI dataset to test whether two distinct representations of the laryngeal muscles can be observed in the cerebellum (see [21] for details on data acquisition). The study was approved by the Central University Research Ethics Committee at the University of Oxford (CUREC, R55787/RE001) in accordance with the regulatory standards of the Code of Ethics of the World Medical Association (Declaration of Helsinki). Twenty participants performed speech movements to localize lips, tongue, and laryngeal activity during vocalization. Participants produced non-linguistic utterances overtly, articulating silently, using am isolated vowel, or as covert speech. The LMC was then localized using a factorial model comparing overt speech and vowel production with silent articulation and covert speech. See [21] for a detailed description of the functional paradigm and analysis.”

**Figure 3:**
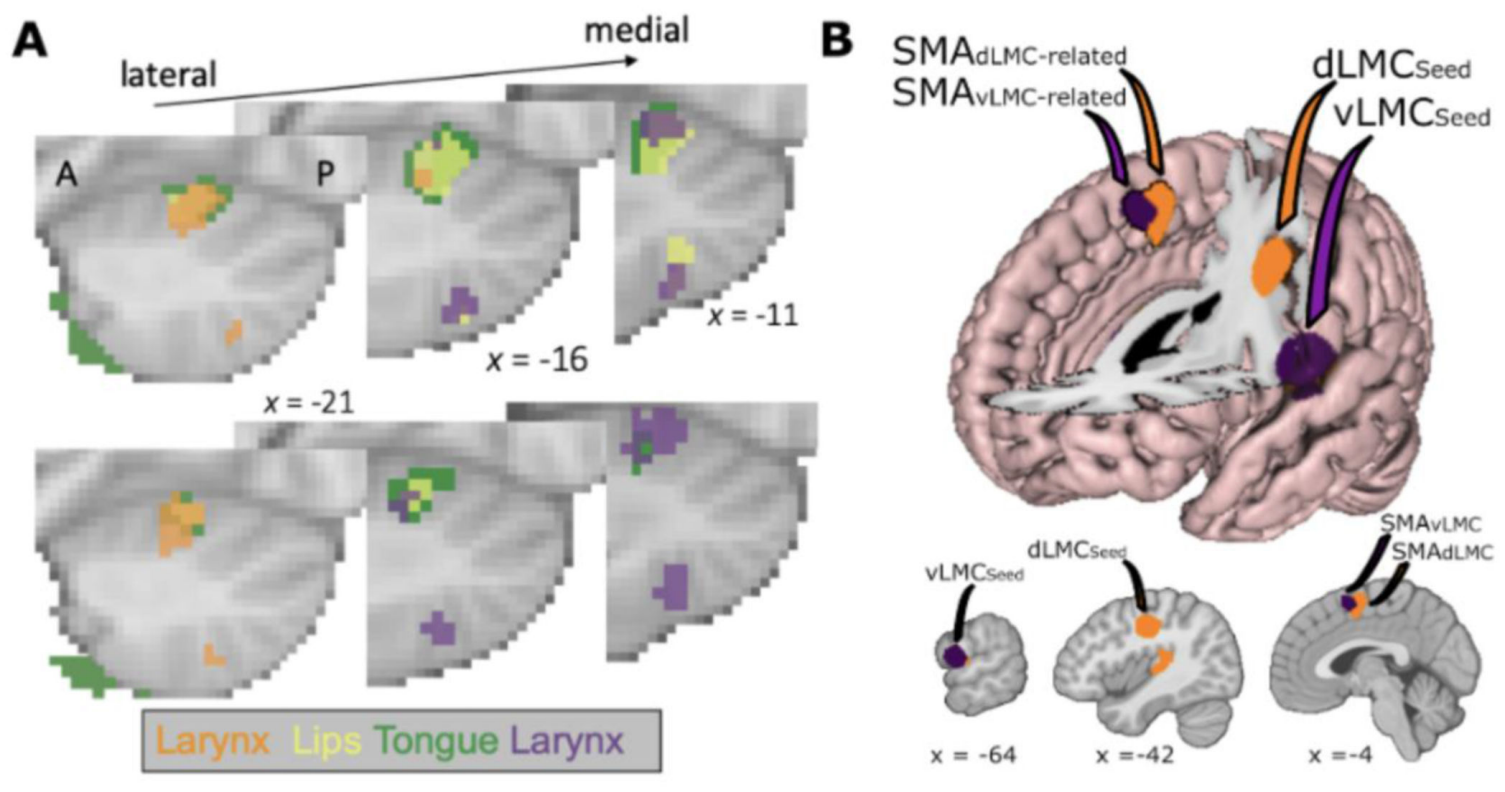
Initial evidence for laryngeal motor network somatotopy. A) Cerebellar task activations during movement of the lips, the tongue and during larynx activity. Shown are sagittal slices of the left hemisphere (A-P: anterior-posterior). Larynx activity is shown in orange and purple to indicate dLMC- and vLMC-related activation, though we note that these are correlated activations derived from the same contrast. Top: Binarized group-level task activations (voxel-wise threshold z > 4, n = 20). Bottom: Binarized overlap maps (individual maps: voxel-wise threshold of z > 3.1, overlap map: thresholded at n > 10 participants). B) Results of ALE meta-analysis from the two LMC seed regions displayed on the MNI152 atlas brain. Top: The surface brain is digitally transected sagittally at x=0, axially at z=10, and coronally with an oblique slice following the precentral gyrus. Bottom: Sagittal slices transecting the two see regions and the SMA. The dLMC-related supplementary motor area (orange) is posterior to the vLMC-related supplementary motor area (purple) in line with the expected somatotopy of this region.

In addition to conventional group-level statistical activation maps, we derived overlap maps of individually thresholded and binarized volumetric maps (see Figure 3A for details of analysis). A larynx-lip-tongue-larynx pattern can be observed along a lateral/anterior-to-medial/posterior axis. The coordinates of these regions are consistent with lobule VI of the posterior cerebellar lobe [130]. Two distinct activations for the larynx can be observed at the group level (Figure 3A, top) as well as in individual participants (Figure 3A, bottom). Activations for the lips and the tongue fall in between the two larynx activations as they do in motor cortex, though at the present resolution these activations are largely overlapping. The dLMC-related activation is observed antero-laterally to the articulators while the vLMC-related activation is observed postero-medially. All activations are in close proximity and within the same anatomical lobule.

Our results are most consistent with one continuous somatotopic map in lobule VI of the cerebellum that contains two distinct laryngeal representations. We note also that additional activations are present at a lower threshold in the remaining lobules, which may reflect additional somatotopic maps [84–88].

### Supplementary motor area

We conducted a meta-analysis of brain imaging studies that activated the dLMC and vLMC to identify brain regions that are co-activated with each larynx area. We searched the BrainMap database [131] for fMRI studies that reported activation within a 5 mm radius sphere of the dLMC (x=-41; y=-16; z=38) or the vLMC (x=-66; y=-4; z=14). This search was performed blind to the tasks being performed by the participants and was concerned only with activation within the seed regions [132]. Coordinate tables in Montreal Neurological Institute (MNI) space were retrieved from the database on 04/04/2020 (see S1 and S2). This searched yielded 512 foci of activation across 29 participant groups for the dLMC, and 294 foci across 19 participant groups for the vLMC. Each set of activation coordinates was analysed using Activation Likelihood Estimation [133–135] using GingerAle software (v3.0.2) with a cluster-level family wise error rate of p<0.01 computed with 5000 permutations. Results were visualized using Mango (v4.1, Research Imaging Institute, UTHSCSA).

The dLMC-related ALE yielded a network of motor and auditory related brain regions including the contralateral dLMC, the superior temporal gyrus (STG), putamen, cerebellum, and the SMA (see Figure 3B and Table 1). The vLMC-related ALE yielded a much more restricted network, as expected from the smaller pool of studies in that analysis, including the contralateral vLMC, the insula, and the SMA. Both ALEs revealed co-activation with the SMA, but at spatially distinct sites. The dLMC-related SMA was posterior to the vLMC-related SMA. This pattern is consistent with the expected somatotopy of this region and with the previously observed network somatotopy between the SMA and motor cortex [128,129].

## Mechanisms of brain network evolution

We have hypothesized that the human brain has evolved not only a dual representation of the laryngeal muscles in motor cortex, but a dual laryngeal motor network to support it. However, this broader characterization of the phenotype raises important questions about how natural selection may act simultaneously on an entire network of brain regions whose functions are strongly interdependent. Among these questions is how the emergence of a novel pathway overcomes strong allometric constraints, for example that dictate the relative volume of grey matter to white [136,137], or how individual neural adaptations can be accommodated within the highly conserved organisation of neocortex [138,139].

There is some debate about the extent to which evolution is able to influence individual brain regions to form an evolutionary mosaic [140,141] as compared to concerted change over the entire brain [142,143]. While brain area size is highly predictable from overall brain size taken at a broad taxonomical scale (e.g., across mammals), individual brain regions violate this trend when examined at a finer taxonomic scale (e.g., across primates), which is a likely driver of inter-species behavioural differences [42,144].

Pairs of functionally related brain structures have correlated sizes across species even after controlling for brain size, indicating that brain networks may evolve together and at least partially independently of other brain structures [140]. Furthermore, natural selection may be capable of acting on individual brain regions and their corresponding networks due to genetic mechanisms that provide independent regulation of brain region sizes [141]. The primate cortical sheet has not expanded uniformly as brain size increased, with the occipital lobe expanding least and the frontal and temporal lobes expanding most, but this pattern is conserved and species differences appear to be the product of brain size [145].

A remarkably analogous instance of network-wide brain evolution is found in the song system of parrots. Strong vocal learning abilities have evolved independently in three lineages of birds, and of these parrots are among the most prodigious vocal learners [50,146]. The avian song system is composed of a series of nuclei, some of which are analogous to structures in the human vocal-motor system including the putamen, motor cortex, and nucleus ambiguus [18,147], and are regulated by specialised patterns of gene expression [148,149]. The parrot brain is unusual in containing two parallel song systems [150]. Nuclei in the parrot song system are composed of a core that is analogous with the song system of other avian vocal learners, and a surrounding shell that forms a rudimentary second song system. The core and shell song systems form parallel networks, however only the core sends direct projections to the brainstem motor nucleus that controls the syrinx (i.e., the analogue to mammalian nucleus ambiguus). Chakraborty & Jarvis (2015) proposed that such a phenotype could arise by mutations that cause the entire network to duplicate as an ensemble, in line with a previous proposal that the avian song system itself may have evolved as a specialization from a pre-existing limb and body motor network [152].

**Figure 4:**
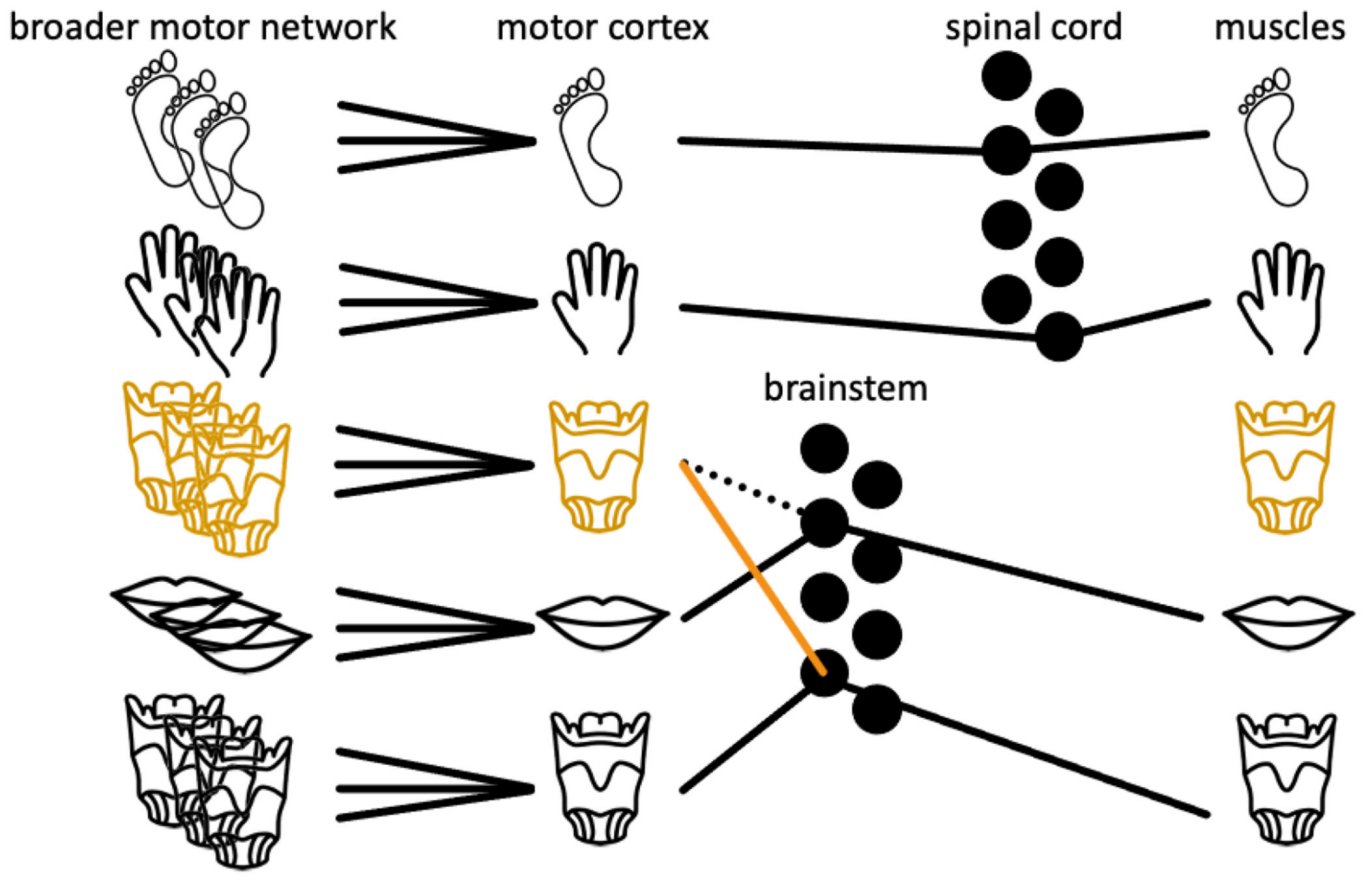
Conceptual depiction of parallel effector-specific circuits feeding from the broader motor network to upper motor neurons in motor cortex and onto lower motor neurons in the brainstem and spinal cord (black circles). We propose that evolutionary changes that add novel downstream targets (orange line) to the efferent motor pathway change the function of the corresponding portion of motor cortex as well as the broader motor networks to which it is connected. The example above depicts a novel projection from a patch of motor cortex to brainstem, which in turn alters the function of the motor network in which it is embedded in to support voice motor control. The dotted line indicates that this patch was previously recruited by a different effector.

We suggest that only a relatively minor change to an existing portion of mammalian motor cortex may have been sufficient to evolve a novel laryngeal motor network in humans. We propose that the emergence of novel efferent pathways to the nucleus ambiguus de facto alters the functional significance not only of these cortical neurons in the motor cortex but also the broader network in which they are embedded (see Figure 4). Given that somatotopic motor networks are defined by the effectors that they control (e.g., M1-hand is that part of motor cortex which projects to hand lower motor neurons in the spinal cord, SMA-hand is that part of the SMA that projects to M1-hand, etc.) modifications to the descending efferent pathways of motor cortex alter the function of corresponding sites throughout motor network. Hence, we propose that the evolution of novel projections from one or both of the LMCs was sufficient for the emergence of vocal motor networks, thereby acquiring novel functions. Such a mechanism would leverage existing long-range connections in the brain, thereby preserving existing allometric relationships between the grey and white matter volumes and overcoming hard barriers for morphological changes.

One mechanism that has been proposed to drive the development of novel laryngeal motor specialisations in humans is the evolution of novel patterns of gene expression in the dLMC and vLMC relative to surrounding cortex [18]. This specialisation includes genes of the slit and plexin family, that encode axon guidance molecules and neuronal growth cone receptors, respectively [153,154]. These genes are likely candidates for a molecular genetic mechanism that may drive the direct projection to nucleus ambiguus in humans. Alternatively, such a specialisation may simply arise as a consequence of the increased proportional size of neocortex. Larger brain regions send more axonal projections and compete more effectively for limited dendritic space [155,156]. For example, among mammals, proportionally larger neocortical size is correlated with deeper penetration of the spinal cord by corticospinal axons, which in turn mediates improved manual dexterity [42,43]. Hence, the increased proportional size of human neocortex alone may have been a driving factor in evolving novel vocal motor networks in humans. As cortical expansion increased the total number of corticobulbar axons, they may have invaded novel territory in the nucleus ambiguus, potentially at the expense of other inputs that mediate unlearned vocalisations, such as the periaqueductal grey [157,158].

We note that the human brain has undergone numerous other large scale structural changes relative to non-human primates [159–164]. The emergence of vocal motor networks is itself not sufficient for the communicative behaviours of humans. Rather, it is part of an ensemble of neural adaptations that support the vocal, auditory, semantic, syntactic, and pragmatic faculties which are needed for speech and language, and which may have separate evolutionary histories [31,32,165]. However, we do suggest that the small-scale modification of the corticobulbar outputs of motor cortex may have had large-scale functional implications for the motor network.

## Summary

We have proposed a novel hypothesis that the dual representation of the laryngeal muscles found in the motor cortex is repeated throughout the motor network. Somatotopic organization is a feature that is found across the network of brain regions that control voluntary movement. Each of these brain regions contains representations of muscle groups following a predictable order based on the plan of the body. These motor regions project preferentially to somatotopically homologous regions (e.g., M1-hand to SMA-hand) to form an extended somatotopic network. Initial evidence suggests that the cerebellum and SMA may also contain dual representations of the larynx, thereby contributing the functions of the cortico-cerebellar and cortico-striatal loops to voice motor control. These findings require further replication and should be extended to other motor regions such as cingulate cortex and the basal ganglia. This hypothesis raises important questions about how adaptations at the level of motor cortex may impact the broader network in which it is embedded. We have also discussed brain evolution in search of a parsimonious mechanism for the emergence of this complex phenotype in the human brain.

## Figures and Tables

**Table 1:** Coordinates of peak likelihoods from ALE meta-analysis for seed regions in the dLMC (upper) and vLMC (lower). Brain regions are listed along with their x, y, z coordinates in MNI stereotaxic space and their Activation Likelihood Estimation scores which provide a relative measure of confidence.

dLMC					
Brain Region	Hemisphere	x	y	z	ALE Value
dLMC [seed]	Left	-42	-16	38	0.125
dLMC	Right	46	-12	38	0.045
SMA	Left	-4	0	56	0.041
Putamen	Right	26	0	4	0.032
Cerebellum	Left	-12	-62	-20	0.032
STG	Left	-60	-14	10	0.027
					
**vLMC**					
Brain Region	Hemisphere	X	y	z	ALE Value
vLMC [seed]	Left	-64	-4	14	0.098
vLMC	Right	66	-4	22	0.024
SMA	Left	-2	8	58	0.025
Right Insula	Right	42	-6	8	0.024
